# An Open-Label Exploratory Clinical Trial Evaluating the Effects of GLS (Coptidis Rhizoma-Evodiae Fructus 2 : 1) on Fibroblast Growth Factor 21 in Patients with Nonalcoholic Fatty Liver Disease

**DOI:** 10.1155/2022/4583645

**Published:** 2022-03-23

**Authors:** Yang Zhang, Jian-Xing Luo, Yan-Ge Li, Hong-Fang Fu, Fang Yang, Xiao-Yu Hu

**Affiliations:** ^1^National Integrative Medicine Clinical Base for Infectious Diseases/Department of Infectious Diseases, Hospital of Chengdu University of Traditional Chinese Medicine, Chengdu 610072, Sichuan Province, China; ^2^Clinical Medical College, Chengdu University of Traditional Chinese Medicine, Chengdu 610072, Sichuan Province, China; ^3^Department of Spleen, Stomach, Liver and Gallbladder, Xin Mi Hospital of Traditional Chinese Medicine, Xinmi 452370, Henan Province, China; ^4^Department of Neurology, Hospital of Chengdu University of Traditional Chinese Medicine, Chengdu 610072, Sichuan Province, China

## Abstract

**Methods:**

In a 12-week, open-label, exploratory clinical trial, 126 NAFLD patients were randomly divided into the GLS group (lifestyle intervention plus GLS) or the polyene phosphatidylcholine (PPC) group (lifestyle intervention plus PPC). Random numbers generated by DPS software were used in combination with opaque, sealed envelopes for allocation concealment. At baseline as well as at the end of the study, anthropometric parameters, glucose, lipids, hepatic enzymes, and FGF 21 were measured, with hepatic fat accumulation assessed by ultrasound (US) and US-based controlled attenuation parameter (CAP).

**Results:**

119 patients completed the study. Baseline parameters did not significantly differ between the two groups (*P* > 0.05). Compared with PPC, GLS decreased more significantly in hepatic fat accumulation, body weight index, waist circumference, waist-to-hip ratio, serum glucose, total cholesterol, triglyceride, low-density lipoprotein cholesterol, alanine transaminase, aspartate transaminase, gamma-glutamyl transferase, and FGF 21 (*P* < 0.05). The effects of GLS on waist circumference, waist-to-hip ratio, CAP, and gamma-glutamyl transferase (GGT) were positively correlated with serum FGF 21 (*r* = 0.343, 0.342, 0.315, and 0.374, respectively, *P* < 0.05). The GGT and FGF-21 changes were also confirmed by multiple linear regression analysis (B, 0.777; 95% CI: 0.307–1.247, *P* < 0.05).

**Conclusion:**

GLS has a significant hepatoprotective effect on NAFLD patients, causing a decrease in FGF-21 secretion in response to the damage itself.

## 1. Introduction

Nonalcoholic fatty liver disease (NAFLD) refers to chronic hepatic damage induced by overnutrition, insulin resistance (IR), and related metabolic disorders. Globally, NAFLD affects approximately 1.8 billion people with a prevalence of 20–30%, of which more than 50% of individuals are overweight or obese [1, 2]. In addition to developing severe liver disease, NAFLD correlates with metabolic syndrome and atherosclerosis [3]. Although changing a poor lifestyle and bariatric surgery have proven effective, most patients still favor medication for compliance and trauma concerns [4]. However, there is currently no approved drug.

Coptidis Rhizoma (Chinese name: Huang-Lian; CR) and Evodiae Fructus (Chinese name: Wu-Zhu-Yu; EF) are classic herb pairs in traditional Chinese medicine (TCM) formulas, which are often used in compatibility. Different compatibility ratios of CR-EF are not the same in terms of efficacy focus, usage, dosage, and clinical application. The most commonly used formulas are “Zuojin wan (CR-EF 6 : 1)” and subsequent derivations of “Zhulian san (CR-EF 5 : 2),” “Ganlu san (GLS; CR-EF 2 : 1),” “Biantong wan (CR-EF 1 : 1),” and “Fanzuojin wan (CR-EF 1 : 6).” Modern studies have confirmed that these formulas cover a wide pharmacological spectrum, including antidepressant, anti-inflammatory, anticancer, antibacterial, and antioxidant effects [5–7]. At present, we have not retrieved the CR-EF in the treatment of NAFLD. CR-contained TCM formulas and the chemical constituents of CR gain greater attention for their potential for treating NAFLD [8–10], while EF gains less attention despite EF increasing energy consumption by inducing heat production and loss [11]. Originally recorded by Sheng Ji Zong Lu (a comprehensive Song Dynasty book of TCM), GLS was used to treat NAFLD patients in the Hospital of Chengdu University of TCM. The results demonstrated that GLS significantly attenuated body weight, glucose and lipid metabolism disorders, and hepatic damage in NAFLD patients.

Hepatokines are proteins secreted by hepatocytes, which play an important role in regulating the metabolic process. In particular, fibroblast growth factor (FGF) 21 is considered a potential therapeutic target for a variety of chronic human diseases, including obesity and type 2 diabetes mellitus [12]. However, numerous studies suggest that serum FGF 21 is positively correlated with the severity and progression of NAFLD [13, 14], while additional studies indicate that high serum FGF 21 concentration is an independent predictor of NAFLD in adults [15]. In view of this interesting phenomenon, we explored the effect of GLS on serum FGF 21 levels in NAFLD patients.

## 2. Materials and Methods

### 2.1. Study Design

An open-label, exploratory clinical trial was conducted at the Department of Infectious Diseases, Hospital of Chengdu University of TCM, from April 2015 to December 2016. For compliance considerations, all eligible patients were assigned to either the GLS group (lifestyle intervention plus GLS) or the polyene phosphatidylcholine (PPC) group (lifestyle intervention plus PPC) for 12 weeks. Hepatic fat accumulation, anthropometric parameters, serum glucose, lipids, hepatic enzymes, and FGF 21 were measured at baseline and at the end of the study. Every four weeks, the patients were followed up by telephone. The study protocol was designed in accordance with the Helsinki Declaration of 1975 and was approved by the ethics committee of the Hospital of Chengdu University of TCM. We obtained written informed consent from patients prior to inclusion in the study.

### 2.2. Diagnostic and Inclusion Criteria

NAFLD was diagnosed based on the following criteria [16]: (1) alcohol consumption <140 g/week for males and <70 g/week for females; (2) absence of hepatitis B, hepatitis C, Wilson's disease, autoimmune diseases, drug-induced liver injury, and a history of total parenteral nutrition; and (3) ultrasonographic examination suggesting hepatic fat accumulation. The inclusion criteria were as follows: (1) 18–65 years of age; and (2) in accordance with the diagnostic criteria of NAFLD.

### 2.3. Exclusion Criteria

Patients were excluded from this study if they had other liver diseases (such as cirrhosis, hepatocellular carcinoma, or decompensated liver disease), other comorbid conditions (such as hypertension, diabetes, neoplastic disease, psychiatric diseases, severe cardiac, or pulmonary disease), received treatment or other herbal drugs in the past two weeks, were pregnant, or breastfeeding. Concerning safety, patients with obvious liver function abnormalities were also excluded (for example, alanine transaminase (ALT), aspartate transaminase (AST), or (TBIL) more than three times the upper limit of normal).

### 2.4. Sample Size Estimate

The sample size was calculated according to previous efficiency data (86% versus 63%), as well as the match ratio (1 : 1), type I error rate (5%), and power (80%). Assuming a 10% rate of loss to follow-up, a total of 126 samples were needed [17].

### 2.5. Randomization and Allocation Concealment

The complete random grouping function of the DPS statistics software was used to generate random sequences according to a 1 : 1 ratio. The random sequences were subsequently sealed in opaque envelopes and kept by two staff members. According to the sequence of patients included, the envelopes were opened sequentially and patients were assigned to the GLS group or PPC group. The individuals who generated and kept the random sequences did not participate in the trial operation, result evaluation, or data statistics.

### 2.6. Intervention

During a two-week run-in period, the research dietician guided patients to maintain a regular diet and physical activity routine and evaluated their compliance. Each patient was given lifestyle intervention plus GLS (8.12 g/d) or PPC (1.368 g/d) for 12 weeks, respectively. GLS was composed of CR and EF, which are displayed in Table 1. Referring to our previous study [18], GLS was made into granules in Sichuan Neo-green Pharmaceutical Technology Development Co., Ltd. The composition of a box of granules (8.12 g) was the same as that of 75 g of raw herbs (i.e., the daily dose per patient). The doses of GLS and PPC were both conventional clinical doses. The 12-week study period was determined by previous literature [19] and our observations, as taking TCM for 12 weeks improved clinical symptoms, blood lipids, liver enzymes, and liver ultrasound characteristics in NAFLD patients.

Both groups were advised to follow an energy-balanced diet and recommended physical activity [20]. Diet composition consisted of carbohydrates (60% to 65%), fat (25%), and protein (15% to 20%). It was also recommended to reduce the intake of sugary drinks, saturated fats, and trans-fats and increase dietary fiber. For patients with a BMI ≥24.0 kg/m^2^, daily caloric intake decreased by 500 to 1000 kcal. All patients are advised to perform moderate aerobic exercise ≥40 min, 4 times a week.

Patients underwent nutritional counseling every 4 weeks by the same dietician during the study. At every visit, the patients were encouraged to continue treatment. Patient adherence to GLS was assessed using patient completed logs, while adherence to PPC was assessed by counting the capsules returned by patients. The patient registered the type and duration of the exercise in a log. Less than 80% of full compliance in each component of the intervention was defined as poor compliance.

### 2.7. Laboratory Assays

Patient's laboratory data were uniformly assayed at the Laboratory Department, Hospital of Chengdu University of TCM. Biochemical parameters were measured by a colorimetric method (Automatic Analyzer 7170A, Hitachi, Japan), including fasting serum level of glucose, lipid (total cholesterol (TC), triglyceride (TG), low-density lipoprotein cholesterol (LDL-C), high-density lipoprotein cholesterol (HDL-C)), hepatic enzymes (alanine transaminase (ALT), aspartate transaminase (AST), and gamma-glutamyl transferase (GGT)). Serum FGF 21 levels were quantified using enzyme-linked immunosorbent assay kits (CUSABIO, Barksdale, DE, USA).

### 2.8. Assessment of Hepatic Fat Accumulation

**Figure 1:**
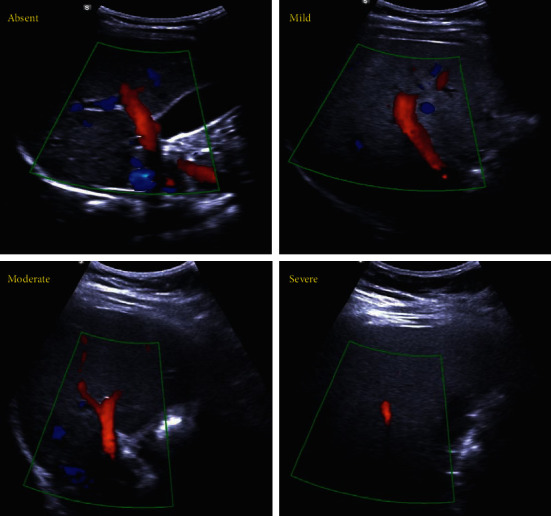
The severity of hepatic fat accumulation assessed by ultrasound.

The degree of hepatic fat accumulation was qualitatively and quantitatively analyzed. The former was completed by a trained US technician using a B-US (ACCUVIX A30, Samsung, Korea). The degree of fat accumulation was graded as mild, moderate, or severe according to the appearance of the liver echotexture, hepatic echo penetration, clarity of the hepatic blood vessels and bile ducts, and the liver diaphragm differentiation in echo amplitude [21] (Figure 1). The latter was completed by a trained and certified nurse using a FibroScan 502 Touch model (Echosens, Paris, France) equipped with an M probe. The measurement of controlled attenuation parameters (CAP) was only performed via the M probe, whose theoretical depth of detection was within 3 cm^3^ of liver tissue from 2.5 cm to 6.5 cm subcutaneously, with the frequency fixed at 3.5 M Hz. The FibroScan examination procedure has been detailed previously [22].

### 2.9. Outcomes

The primary outcome of this study was CAP value at week 12. Secondary outcomes were the improvement in anthropometric parameters, glucose and lipid metabolic parameters, hepatic enzymes, FGF 21, and hepatic fat accumulation assessed by US.

### 2.10. Statistical Analysis

Efficacy analysis was based on the per protocol set (PPS), which included all patients who completed the study and who did not violate any of the inclusion/exclusion criteria or deviate from the protocol in a way that could affect the outcome of the study. The safety analysis set consisted of all patients who received the study drug, regardless of whether they completed the study [23].

The quantitative data were described by the mean ± SD. The *χ*^2^ test and the Mann–Whiney *U* test were used to evaluate the differences in sex and US-assessed hepatic fat accumulation between the two groups. An independent sample *t*-test and a Wilcoxon rank sum test were performed to compare anthropometric parameters, glucose and lipid metabolic parameters, hepatic enzymes, CAP value, and FGF 21 between the two groups at baseline. The change in each parameter was calculated as the difference between the before and after treatment values. At the end of the study, a one-factor analysis of covariance (ANCOVA) using each parameter change as the dependent variables and the baseline values as the covariates was performed to compare the differences in the effects of GLS and PPC. The Pearson and Spearman methods were used to analyze the correlation between the changes in body mass index (BMI), waist circumference (WC), waist-to-hip ratio (WHR), glucose, lipids, haptic enzymes, fat accumulation, and FGF 21 change. Explanatory variables identified as significant in bivariate analysis were subsequently entered into a multiple linear regression model, with FGF 21 as the dependent variable. All statistical tests were two-tailed, and a significance level (P) of 0.05 was used. The statistical tests were performed using the Statistical Package for the Social Sciences (SPSS) version 21.0 (SPSS Inc., Chicago, IL, United States).

### 2.11. Patient or Public Involvement

Patients and members of the public were not involved in the design of this study.

## 3. Results

### 3.1. Patients

**Figure 2:**
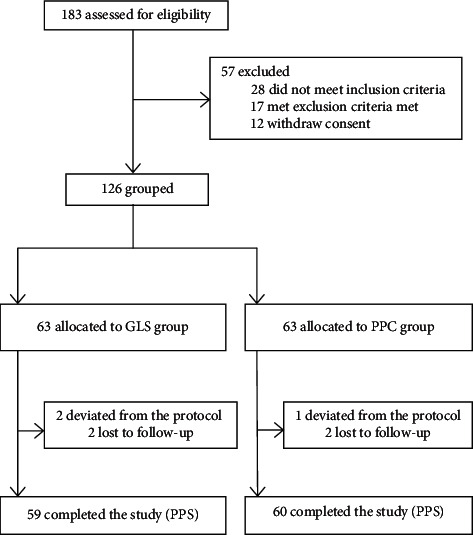
Patients' disposition during the study. PPS, per protocol set.

Figure 2 shows the patients' disposal during the study, with 126 eligible patients recruited from 183 NAFLD patients. Among them, three patients were excluded (protocol deviation) and four patients were removed partway for poor compliance. The PPS population included 119, with 59 in the GLS group and 60 in the PPC group.

### 3.2. Baseline Characteristics

There was no significant difference in age, sex, height, hip circumference, anthropometric parameters, clinical, and laboratory data at baseline between the two groups (*P* > 0.05; Table 2).

### 3.3. Effects of GLS on the Severity of Hepatic Fat Accumulation

**Figure 3:**
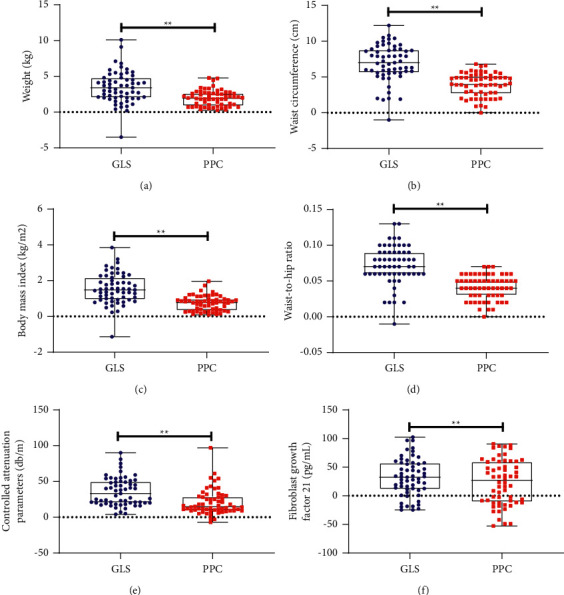
Boxplots and dot plots of non-biochemical parameters changes between the two groups. GLS, ganlusan; PPC, polyene phosphatidylcholine; ^*∗∗*^the *P* value representing a comparison of parameters changes between the two groups of is less than 0.01.

At the end of the study, the CAP value was significantly decreased in the GLS group compared with the PPC group (Table 2 and Figure 3). Following treatment, hepatic fat accumulation assessed by US in the GLS group was significantly less than that in the PPC group (Table 3).

### 3.4. Effects of GLS on Anthropometric Parameters

After 12 weeks of intervention, weight, BMI, WC, and WHR presented more significant decreases in the GLS group than in the PPC group (Table 2 and Figure 3).

### 3.5. Effects of GLS on Glucose and Lipid Metabolism Parameters

**Figure 4:**
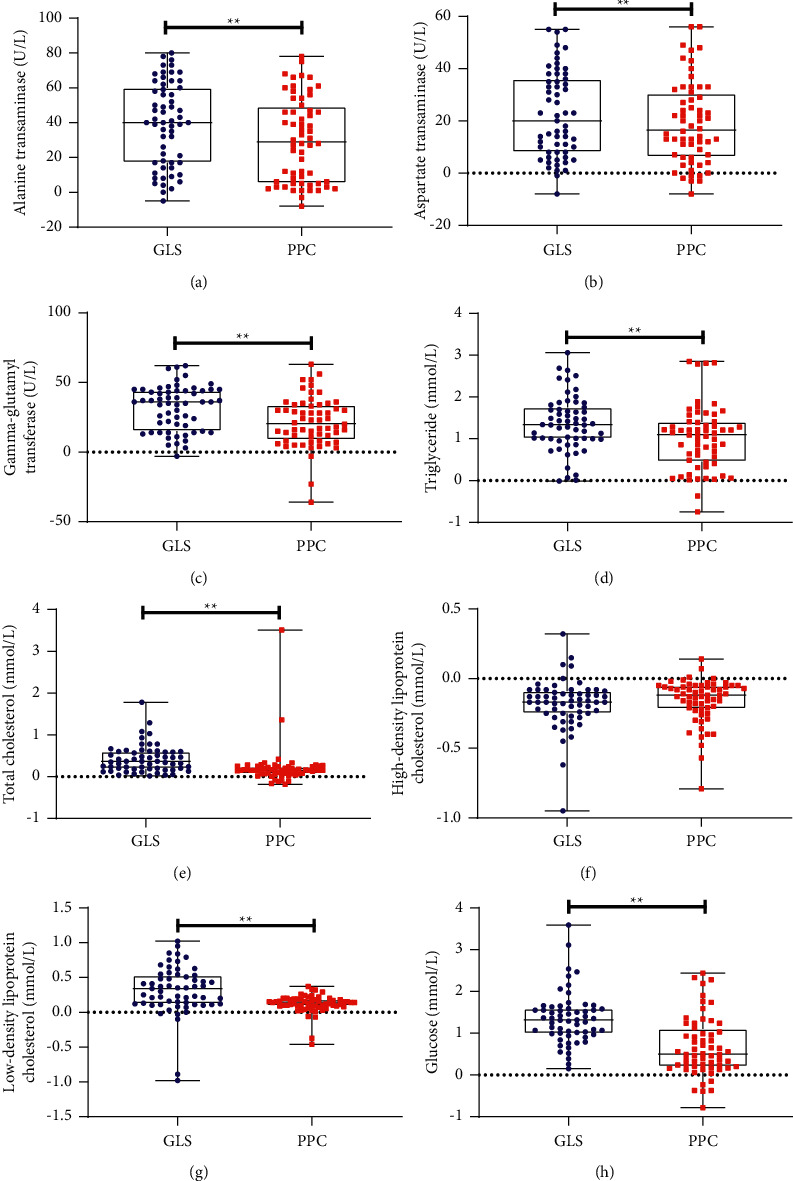
Boxplots and dot plots of biochemical parameters changes between the two groups. GLS, ganlusan; PPC, polyene phosphatidylcholine; ^*∗∗*^the *P* value representing a comparison of parameters changes between the two groups of is less than 0.01.

Following treatment, serum TC, TG, LDL-C, and glucose levels decreased more significantly in the GLS group than in the PPC group (Table 2). However, there was no significant difference in the change of serum HDL-C levels between the two groups (Table 2 and Figure 4).

### 3.6. Effects of GLS on Hepatic Enzymes Markers

Compared with the PPC group, the GLS group had more ALT, AST, and GGT decline following treatment (Table 2 and Figure 4).

### 3.7. Effects of GLS on Serum FGF 21 Levels

At week 12, serum FGF 21 levels decreased more significantly in the GLS group than those in the PPC group (Table 2 and Figure 3).

### 3.8. Correlation between Serum FGF 21 and Other Parameter Changes

**Figure 5:**
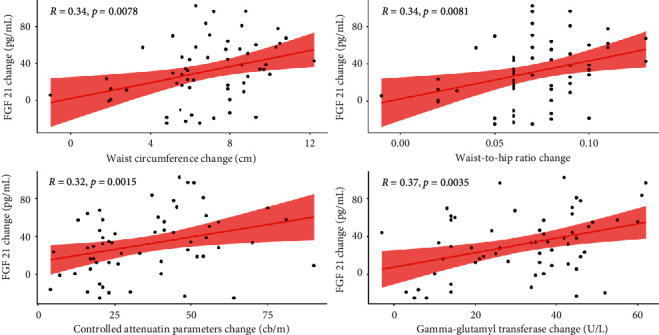
The plot of Pearson correlation of FGF 21 change with the changes in waist circumference, waist-to-hip ratio, controlled attenuation parameters, and gamma-glutamyl transferasec in polyene phosphatidylcholine group.

Correlation data from the GLS group demonstrated that the effects of GLS on WC, WHR, CAP, and GGT were positively correlated with serum FGF 21 (*r* = 0.343, 0.342, 0.315, and 0.374, respectively) (*P* < 0.05). The GGT and FGF 21 changes were also confirmed by multiple linear regression analysis (B, 0.777; 95% CI: 0.307–1.247) (*P* < 0.05). However, based on the PPC group data, no linear correlation was found between serum FGF 21 and other parameter changes (*P* > 0.05). The results are shown in Table 4 and Figure 5.

### 3.9. Safety

No serious adverse events occurred, and all adverse reactions are shown in Table 5.

## 4. Discussion

In this study, GLS (CR-EF 2 : 1) exhibited a positive therapeutic effect on NAFLD, with GLS improving multiple pathological processes such as excess energy, glucose and lipid metabolism disorders, and hepatic injury.

TCM has a long history as a key alternative treatment, with TCM including CR and EF predominately used clinically in compatibility (namely formulas) to achieve synergy and detoxification. The compatibility of CR and EF remains a key focus of research. Peng et al. [24] determined that different ratios of CR-EF inhibited the growth of human gastric carcinoma cells and induced their apoptosis, with the strongest effect being 6 : 1. Moreover, Zhao et al. [25] investigated the effects of CR-EF in different ratios on catecholamine secretion induced by acetylcholine in cultured bovine adrenal medullary cells. Interestingly, CR-EF 6 : 1 and 1 : 6 displayed the opposite effects. Qian et al. [26] explored changes of CR-EF *in vivo* from the perspective of pharmacokinetics, and revealed that the absorption, elimination, and systemic exposure level of 12 alkaloids were mainly influenced by the ratio of CR-EF. These findings would help to enhance our understanding of the efficacy focus and internal mechanism of CR-EF in different ratios. In the absence of CR and EF used alone or in compatibility for NAFLD, our results supported GLS as an effective formula.

The hallmark of NAFLD is fat (TG) accumulation in the hepatocytes, which is mainly attributed to IR and excess free fatty acids (FFA) uptake by the liver. In their healthy state, approximately 60% of the hepatic fat in human subjects is derived from FFA produced by lipolysis of adipose tissue [23]. NAFLD is closely associated with overnutrition, with increased fat mass and insulin resistance (IR) leading to higher lipolysis along with hepatic FFA concentrations [24].

Recently, increased attention has been paid to the so-called organokines, proteins with autocrine, paracrine, or endocrine activities. Of them, adipokines (fat-derived), myokines (skeletal muscle-derived), and hepatokines are predominantly produced by the liver. FGF 21, which belongs to hepatokines, plays a significant role in the pathogenesis of NAFLD. In the FGF 21 signaling pathway, FGF 21 is recruited to the extracellular surface of the plasma membrane by the *β*-Klotho (its obligate coreceptor) [27]. The *β*-Klotho/FGF 21 receptor complex specifically interacts with homologous receptors (FGFR1c, FGFR2c, or FGFR3c), enabling downstream FGFR signaling transduction by pathways such as mitogen-activated protein kinase and the AKT signaling network [28, 29]. After entering the systemic circulation, FGF 21 can integrate metabolism across the liver, adipose tissue, skeletal muscle, pancreas, and other metabolic organs by controlling expression of transcriptional programs that shape cellular phenotype and tissue metabolic function of the target organs, ultimately exerting antiobesity, antidiabetic, antihyperlipidemic, and anti-NAFLD effects in rodents and primates [30].

However, many clinical studies have demonstrated that increased serum FGF 21 predicts the occurrence of NAFLD and has been positively correlated with metabolic disorders and hepatic damage [31, 32]. Hence, FGF 21 appears to have a paradoxical effect on the metabolic regulation between animals and humans. Some scholars believe that FGF resistance exists in NAFLD patients, which is due to oxidative damage and chronic inflammation inhibiting the expression of *β*-klotho and FGFR, resulting in increased compensatory FGF 21 synthesis and secretion [33–36]. Reportedly, certain anti-NAFLD treatments may lead to a significant decrease in FGF 21 by improving its resistance [37, 38].

In addition, new perspectives help to understand this phenomenon. Fisher et al. [35] and Liu et al. [36] determined that FGF 21 played a role in cell reparation to resist cytotoxicity and helped to maintain metabolic homeostasis through the hormonal pathways. In FGF 21 knockout mice, hepatic damage and mortality caused by excessive paracetamol significantly increased, while the recovery of recombinant FGF 21 was mostly reversed. Their findings suggested that acetaminophen overdose raised FGF 21, which might protect the liver from drug-induced hepatotoxicity in some ways [39].

Similarly, the increase in FGF 21 is also an important self-help behavior for the liver in the face of other attacks such as fat. Correlation data from the GLS group showed that the effects of GLS on WC, WHR, CAP, and GGT were positively correlated with serum FGF 21 (*r* = 0.343, 0.342, 0.315, and 0.374, respectively) (*P* < 0.05). GLS seemed feasible to reduce FGF 21 secretion by lowering the CAP value. CAP is a new parameter redefined by the ultrasonic attenuation principle, which is mainly used for the quantitative detection of human liver fat. The volume measured by CAP is 100 times that of liver biopsy tissue. CAP is able to evaluate hepatic fat accumulation noninvasively and quantitatively, showcasing positive diagnostic value for hepatic fat in clinical trials [40, 41].

This study used an open-label exploratory clinical trial. Due to the particularities of the study, such as the different dosage forms of GLS and PPC, it was difficult to blind patients or interveners, so we designed an open-label trial. Exploratory clinical trials are conducted in an environment of high drug candidate turnover, low market approval rates, and an alarming waste of research and development fund, and conducted exploratory studies on drugs with clinical experience, so as to guide the entry or abandonment of subsequent confirmatory clinical trials. According to medical observations, GLS may be beneficial to NAFLD without an available drug.

Our results exhibited GLS as a quite effective formula, especially in reducing hepatic fat accumulation. As likely, its hepatoprotective effect led to reduced secretion of FGF 21 in response to liver attacks. These new findings help to understand the relationship between FGF 21 and NALFD and the clinical application of GLS. As a single-center, open-label, exploratory study, the effect of GLS on FGF 21 in NAFLD patients and its mechanism need to be further confirmed. Additionally, the optimal ratio between CR and EF in treating NAFLD also needs to be discussed.

## 5. Conclusion

GLS has a significant hepatoprotective effect on NAFLD patients, causing a decrease in FGF 21 secretion in response to the damage itself.

## Figures and Tables

**Table 1:** The list of raw herbs composing Ganlu san.

Chinese name	Full scientific name	Medicinal parts	Origin	Dose of Chinese herbal piece (g)	Dose after extraction (g)	Content of formula granule (mg/g)
Huang Lian	Rhizoma Coptidis	Rhizoma	Sichuan	50	4.55	163.2 (berberine) 21.6 (epiberberine) 36.9 (coptisine) 42.5 (palmatine)
Wu Zhu Yu	Evodiae Fructus	Fruit	Sichuan	25	3.57	1.33 (evodiamine and rutaecarpine)
Total				75	8.12	

**Table 2:** Comparison of baseline characteristics and parameters changes after treatment.

Variables	GLS (*n* = 59)			PPC (*n* = 60)			F	*P* value
	Baseline	End	Change	Baseline	End	Change		
Weight (kg)	71.04 ± 6.17	67.59 ± 6.43	3.45 ± 2.22	69.31 ± 7.17	67.36 ± 7.03	1.95 ± 1.10	20.226	0.000
Waist circumference (cm)	84.34 ± 4.73	77.41 ± 5.06	6.93 ± 2.58	83.98 ± 5.32	80.06 ± 5.26	3.92 ± 1.62	58.265	0.000
Body mass index (kg/m^2^)	26.70 ± 1.04	25.17 ± 1.12	1.54 ± 0.84	26.42 ± 1.01	25.68 ± 1.06	0.74 ± 0.42	39.725	0.000
Waist-to-hip ratio	0.88 ± 0.05	0.81 ± 0.06	0.07 ± 0.03	0.88 ± 0.54	0.84 ± 0.54	0.04 ± 0.02	57.231	0.000
Controlled attenuation parameters (db/m)	271.51 ± 27.89	235.88 ± 28.66	35.63 ± 19.70	270.48 ± 24.06	249.70 ± 20.32	20.78 ± 17.78	21.574	0.000
Fibroblast growth factor 21 (pg/mL)	130.80 ± 28.29	98.82 ± 17.64	31.97 ± 32.69	135.02 ± 32.12	111.49 ± 21.65	23.53 ± 39.34	12.073	0.001
Alanine transaminase (U/L)	73.51 ± 28.65	34.10 ± 10.76	39.41 ± 23.79	73.13 ± 31.41	43.17 ± 15.41	30.02 ± 24.04	23.373	0.000
Aspartate transaminase (U/L)	53.10 ± 18.88	30.66 ± 10.89	22.44 ± 16.50	55.07 ± 20.95	35.98 ± 12.23	19.08 ± 15.86	7.224	0.008
Gamma-glutamyl transferase (U/L)	75.39 ± 21.63	43.66 ± 15.45	31.73 ± 15.99	74.72 ± 22.75	53.20 ± 18.08	21.52 ± 17.83	17.752	0.000
Triglyceride (mmol/L)	3.23 ± 0.76	1.86 ± 0.35	1.37 ± 0.67	3.11 ± 0.94	2.10 ± 0.47	1.01 ± 0.76	7.659	0.007
Total cholesterol (mmol/L)	5.28 ± 0.31	4.85 ± 0.44	0.43 ± 0.33	5.25 ± 0.31	5.04 ± 0.49	0.21 ± 0.48	2.833	0.005
High-density lipoprotein cholesterol (mmol/L)	1.07 ± 0.30	1.25 ± 0.28	−0.18 ± 0.18	1.01 ± 0.28	1.17 ± 0.25	−0.16 ± 0.16	0.560	0.456
Low-density lipoprotein cholesterol (mmol/L)	3.11 ± 0.45	2.79 ± 0.41	0.31 ± 0.35	3.08 ± 0.31	2.96 ± 0.29	0.12 ± 0.13	20.541	0.000
Glucose (mmol/L)	6.24 ± 0.60	4.89 ± 0.11	1.35 ± 0.63	6.21 ± 0.70	5.53 ± 0.82	0.68 ± 0.71	30.874	0.000

All the values are expressed as the mean ± SD. The *P*values compare the parameters changes between the two groups.

**Table 3:** Ultrasound-assessed the severity of hepatic fatty accumulation at baseline and the end of the study (*n*, %).

	Baseline				*Z*	*P*	End				*Z*	*P*
	Absent	Mild	Moderate	Severe			Absent	Mild	Moderate	Severe		
GLS (*n* = 59)	0 (0)	18 (30.5)	20 (33.9)	21 (35.6)	−0.090	0.928	21 (35.6)	25 (42.4)	8 (13.6)	5 (8.5)	−2.634	0.008
PPC (*n* = 60)	0 (0)	16 (26.7)	24 (40.0)	20 (33.3)			8 (13.3)	30 (50.0)	15 (25.0)	7 (11.7)		

The *P* values compare the severity of hepatic fatty accumulation at baseline and the end of the study between the two groups.

**Table 4:** Correlation between serum FGF 21 and other parameters changes.

Variables	GLS (*n* = 59)		PPC (*n* = 60)	
	Pearson correlation		Pearson/Spearman correlation	
	FGF 21 change (variable)		FGF 21 change (variable)	
	*r*	*P*	*r*	*P*
Weight change	0.000	0.999	0.143	0.275
Body mass index change	−0.028	0.833	0.151	0.249
Waist circumference change	0.343	0.008	0.065	0.621
Waist-to-hip ratio change	0.342	0.008	0.065	0.620
Controlled attenuation parameters change	0.315	0.015	0.158	0.229
Alanine transaminase change	−0.015	0.913	−0.072	0.585
Aspartate transaminase change	−0.077	0.564	−0.099	0.454
Gamma-glutamyl transferase change	0.374	0.004	−0.072	0.587
Triglyceride change	0.151	0.254	0.137	0.294
Total cholesterol change	0.016	0.906	0.055	0.676
High-density lipoprotein cholesterol change	−0.144	0.278	0.194	0.138
Low-density lipoprotein cholesterol change	0.232	0.077	−0.083	0.530
Glucose change	0.029	0.830	−0.081	0.539

^a^The *P*-values compare the significance levels of linear correlation between serum FGF 21 and other parameters changes.

**Table 5:** All adverse events during treatment (*n*, %).

Variable	GLS (*n* = 59)	PPC (*n* = 60)	*χ* ^2^	*P* value
Hypodynamia	0 (0)	1 (1.7)	0.000	1.000
Anorexia	1 (1.7)	3 (5)	0.242	0.623
Irritability	0 (0)	1 (1.7)	0.000	1.000
Diarrhea	1 (1.7)	1 (1.7)	0.000	1.000
Gastric distension	2 (3.4)	1 (1.7)	0.000	1.000

## Data Availability

The data used to support the findings of this study are included within the article.

## References

[1] Younossi Z., Anstee Q. M., Marietti M. (2018). Global burden of NAFLD and NASH: trends, predictions, risk factors and prevention. *Nature Reviews Gastroenterology & Hepatology*.

[2] Younossi Z. M., Koenig A. B., Abdelatif D., Fazel Y., Henry L., Wymer M. (2016). Global epidemiology of nonalcoholic fatty liver disease-Meta-analytic assessment of prevalence, incidence, and outcomes. *Hepatology*.

[3] Manne V., Handa P., Kowdley K. V. (2017). Pathophysiology of nonalcoholic fatty liver disease/nonalcoholic steatohepatitis. *Clinics in Liver Disease*.

[4] Romero-Gómez M., Zelber-Sagi S., Trenell M. (2017). Treatment of NAFLD with diet, physical activity and exercise. *Journal of Hepatology*.

[5] Wang Q.-S., Ding S.-L., Mao H.-P., Cui Y.-L., Qi X.-J. (2013). Antidepressant-like effect of ethanol extract from Zuojin Pill, containing two herbal drugs of Rhizoma Coptidis and Fructus Evodiae, is explained by modulating the monoaminergic neurotransmitter system in mice. *Journal of Ethnopharmacology*.

[6] Wang Q.-S., Cui Y.-L., Dong T.-J., Zhang X.-F., Lin K.-M. (2012). Ethanol extract from a Chinese herbal formula, “Zuojin Pill”, inhibit the expression of inflammatory mediators in lipopolysaccharide-stimulated RAW 264.7 mouse macrophages. *Journal of Ethnopharmacology*.

[7] Qiu C., Cui Y. L., Qi X. J., Jiang H. L., Wang Q. S. (2015). Advance in modern studies on compatibility of coptidis rhizoma and Evodiae fructus. *Zhongguo Zhongyao Zazhi*.

[8] Wang J., Wang L., Lou G. H. (2019). Coptidis Rhizoma: a comprehensive review of its traditional uses, botany, phytochemistry, pharmacology and toxicology. *Pharmaceutical Biology*.

[9] Huang Z., Xu X., Lu F. (2013). Jiao tai wan attenuates hepatic lipid accumulation in type 2 diabetes mellitus. *Evid Based Complement Alternat Med*.

[10] Ohta Y., Sasaki E., Nishida K. (1998). Inhibitory effect of Oren-gedoku-to (Huanglian-Jie-Du-Tang) extract on hepatic triglyceride accumulation with the progression of carbon tetrachloride-induced acute liver injury in rats. *Journal of Ethnopharmacology*.

[11] Kobayashi Y., Nakano Y., Kizaki M., Hoshikuma K., Yokoo Y., Kamiya T. (2001). Capsaicin-like anti-obese activities of evodiamine from fruits of evodia rutaecarpa, a vanilloid receptor agonist. *Planta Medica*.

[12] Degirolamo C., Sabbà C., Moschetta A. (2016). Therapeutic potential of the endocrine fibroblast growth factors FGF19, FGF21 and FGF23. *Nature Reviews Drug Discovery*.

[13] Yilmaz Y., Eren F., Yonal O. (2010). Increased serum FGF21 levels in patients with nonalcoholic fatty liver disease. *European Journal of Clinical Investigation*.

[14] Li H., Fang Q., Gao F. (2010). Fibroblast growth factor 21 levels are increased in nonalcoholic fatty liver disease patients and are correlated with hepatic triglyceride. *Journal of Hepatology*.

[15] Li H., Dong K., Fang Q. (2012). High serum level of fibroblast growth factor 21 is an independent predictor of non-alcoholic fatty liver disease: a 3-year prospective study in China. *Journal of Hepatology*.

[16] Jian-gao F., Chinese Liver Disease Association (2010). Guidelines for management of nonalcoholic fatty liver disease: an updated and revised edition. *Zhonghua Gan Zang Bing Za Zhi*.

[17] Cohen J. (1988). *Statistical Power Analysis for the Behavioral Sciences*.

[18] Zhang Y., Wang Y. T., Luo J. X. (2019). Effect of wsp, a chinese herbal formula, on th17/treg ratio and hbeag seroconversion in telbivudine-treated hbeag-positive chronic hepatitis b patients with high baseline alt levels (20–30 times the uln). *Evidence-based Complementary and Alternative Medicine*.

[19] Fan J. G., Shanghai Multicenter Clinical Cooperative Group of Danning Pian Trial (2004). Evaluating the efficacy and safety of Danning Pian in the short-term treatment of patients with non-alcoholic fatty liver disease: a multicenter clinical trial. *Hepatobiliary and Pancreatic Diseases International*.

[20] Committee of Hepatology (2019). Expert recommendations on standardized diagnosis and treatment for fatty liver disease in China (2019 revised edition). *Zhonghua Gan Zang Bing Za Zhi*.

[21] Graif M., Yanuka M., Baraz M. (2000). Quantitative estimation of attenuation in ultrasound video images. *Investigative Radiology*.

[22] de Lédinghen V., Vergniol J. (2008). Transient elastography (FibroScan). *Gastroentérologie Clinique et Biologique*.

[23] Kay G. G., Maruff P., Scholfield D. (2012). Evaluation of cognitive function in healthy older subjects treated with fesoterodine. *Postgraduate Medicine*.

[24] Peng Q. X., Yang D. J., Shi J., Cai H. B., Mo Z. X. (2011). Proportion of Coptidis rhizoma and Evodiae fructus in the compound preparation: its effect in inducing apoptosis of SGC-7901 cells. *Nan Fang Yi Ke Da Xue Xue Bao*.

[25] Zhao F. R., Mao H. P., Zhang H. (2010). Antagonistic effects of two herbs in Zuojin Wan, a traditional Chinese medicine formula, on catecholamine secretion in bovine adrenal medullary cells. *Phytomedicine: International Journal of Phytotherapy and Phytopharmacology*.

[26] Qian P., Zhang Y.-B., Yang Y.-F., Xu W., Yang X.-W. (2017). Pharmacokinetics studies of 12 alkaloids in rat plasma after oral administration of zuojin and fan-zuojin formulas. *Molecules*.

[27] Lee S., Choi J., Mohanty J. (2018). Structures of *β*-klotho reveal a “zip code”-like mechanism for endocrine FGF signalling. *Nature*.

[28] Ogawa Y., Kurosu H., Yamamoto M. (2007). *β*Klotho is required for metabolic activity of fibroblast growth factor 21. *Proceedings of the National Academy of Sciences*.

[29] Ornitz D. M., Itoh N. (2015). The fibroblast growth factor signaling pathway. *WIREs Developmental Biology*.

[30] Kharitonenkov A., Wroblewski V. J., Koester A. (2007). The metabolic state of diabetic monkeys is regulated by fibroblast growth factor-21. *Endocrinology*.

[31] Liu J., Xu Y., Hu Y., Wang G. (2015). The role of fibroblast growth factor 21 in the pathogenesis of non-alcoholic fatty liver disease and implications for therapy. *Metabolism Clinical and Experimental*.

[32] Giannini C., Feldstein A. E., Santoro N. (2013). Circulating levels of FGF-21 in obese youth: associations with liver fat content and markers of liver damage. *Journal of Clinical Endocrinology & Metabolism*.

[33] Tucker B., Li H., Long X., Rye K. A., Ong K. L. (2019). Fibroblast growth factor 21 in non-alcoholic fatty liver disease. *Metabolism, ” Metabolism*.

[34] Dushay J., Chui P. C., Gopalakrishnan G. S. (2010). Increased fibroblast growth factor 21 in obesity and nonalcoholic fatty liver disease. *Gastroenterology*.

[35] Fisher F. M., Chui P. C., Antonellis P. J. (2010). Obesity is a fibroblast growth factor 21 (FGF21)-resistant state. *Diabetes*.

[36] Liu J., Xu Y., Hu Y., Wang G. (2015). The role of fibroblast growth factor 21 in the pathogenesis of non-alcoholic fatty liver disease and implications for therapy. *Metabolism Clinical and Experimental*.

[37] Chen Y., Feng R., Yang X. (2019). Yogurt improves insulin resistance and liver fat in obese women with nonalcoholic fatty liver disease and metabolic syndrome: a randomized controlled trial. *American Journal of Clinical Nutrition*.

[38] Qin Y., Zhou Y., Chen S. H. (2015). Fish oil supplements lower serum lipids and glucose in correlation with a reduction in plasma fibroblast growth factor 21 and prostaglandin E2 in nonalcoholic fatty liver disease associated with hyperlipidemia: a randomized clinical trial. *PLoS One*.

[39] Li R., Guo C., Wu X., Huang Z., Chen J. (2017). FGF21 functions as a sensitive biomarker of APAP-treated patients and mice. *Oncotarget*.

[40] Fan J.-G., Farrell G. C. (2009). Epidemiology of non-alcoholic fatty liver disease in China. *Journal of Hepatology*.

[41] Myers R. P., Pollett A., Kirsch R. (2012). Controlled Attenuation Parameter (CAP): a noninvasive method for the detection of hepatic steatosis based on transient elastography. *Liver International*.

